# Discovering functional gene expression patterns in the metabolic network of *Escherichia coli *with wavelets transforms

**DOI:** 10.1186/1471-2105-7-119

**Published:** 2006-03-08

**Authors:** Rainer König, Gunnar Schramm, Marcus Oswald, Hanna Seitz, Sebastian Sager, Marc Zapatka, Gerhard Reinelt, Roland Eils

**Affiliations:** 1 Department of Bioinformatics and Functional Genomics, Institute for Pharmacy and Molecular Biotechnology, University of Heidelberg, 69120 Heidelberg, Germany; 2 Theoretical Bioinformatics, German Cancer Research Center (DKFZ), 69120 Heidelberg, Germany; 3 Institute of Computer Science, University of Heidelberg, 69120 Heidelberg, Germany; 4 Interdisciplinary Center for Scientific Computing, University of Heidelberg, 69120 Heidelberg, Germany

## Abstract

**Background:**

Microarray technology produces gene expression data on a genomic scale for an endless variety of organisms and conditions. However, this vast amount of information needs to be extracted in a reasonable way and funneled into manageable and functionally meaningful patterns. Genes may be reasonably combined using knowledge about their interaction behaviour. On a proteomic level, biochemical research has elucidated an increasingly complete image of the metabolic architecture, especially for less complex organisms like the well studied bacterium *Escherichia coli*.

**Results:**

We sought to discover central components of the metabolic network, regulated by the expression of associated genes under changing conditions. We mapped gene expression data from *E. coli *under aerobic and anaerobic conditions onto the enzymatic reaction nodes of its metabolic network. An adjacency matrix of the metabolites was created from this graph. A consecutive ones clustering method was used to obtain network clusters in the matrix. The wavelet method was applied on the adjacency matrices of these clusters to collect features for the classifier. With a feature extraction method the most discriminating features were selected. We yielded network sub-graphs from these top ranking features representing formate fermentation, in good agreement with the anaerobic response of hetero-fermentative bacteria. Furthermore, we found a switch in the starting point for NAD biosynthesis, and an adaptation of the l-aspartate metabolism, in accordance with its higher abundance under anaerobic conditions.

**Conclusion:**

We developed and tested a novel method, based on a combination of rationally chosen machine learning methods, to analyse gene expression data on the basis of interaction data, using a metabolic network of enzymes. As a case study, we applied our method to *E. coli *under oxygen deprived conditions and extracted physiologically relevant patterns that represent an adaptation of the cells to changing environmental conditions. In general, our concept may be transferred to network analyses on biological interaction data, when data for two comparable states of the associated nodes are made available.

## Background

Over the last 40 years, biochemical investigations have discovered an increasingly consistent image of cellular metabolism (see e.g. [1]). This is especially true for less complex organisms such as *Escherichia coli *[2]. However, this alone provides a rather static image of the cell and thus investigations have been performed to discover cellular adaptation programs in response to changing environments such as nutrient excess, starvation and other stresses [3]. These observations originally followed rather linear interaction and reaction cascades, e.g. by investigating single knock-outs and tediously tracking of transcripts for single genes, or compounds and proteins that may potentially be influenced (see e.g. [4]). However, the advent of DNA microarrays has allowed us to explore a major subset or all genes of an organism under a variety of conditions such as alternative treatments, mutants, developmental stages and time points. For example, the technique enables us to classify tumour samples [5], to define small sets of potential marker genes to distinguish leukemias [6], and to discover regulatory mechanisms [7,8]. E.g., without prior information, the structure and function of the network that regulates the SOS pathway in *E. coli *could be elucidated with transcription profiles [9]. Furthermore, physical and chemical interaction data of proteins have been integrated. Knowledge of protein-protein interaction from high-throughput techniques [10] was applied to analyse gene expression data and revealed novel regulatory circuits [11]. Moreover, interaction knowledge from the biochemical network has been used to support the clustering procedure for gene expression profiles of yeast [12,13].

In the work reported here, we sought to reveal sub-graphs of a biological interaction network that show substantial adaptations when cells transcriptionally respond to a changing environment or treatment. As a case study, we investigated the response of the hetero-fermentative bacterium *E. coli *in response to oxygen deprivation. The regulatory machinery can react on this environmental change in different ways. One basic response changes the catabolism of glucose, switching off or down-regulating the respiratory sub-graphs such as the glyoxylate cycle and switching on the fermentation and production of acid end products (see e.g. [4]). This is supported by several signalling concepts, e.g. by inducing inhibitors for glyoxylate cycle genes, down-regulating glyoxylate cycle genes or activating and up-regulating genes for the fermentation processes. Simple clustering of gene expression data on these metabolic networks can yield sub-graphs that are either stimulated or repressed as we showed previously for the tryptophan biosynthesis of tryptophan treated cells [14]. With this method we were able to find an expression pattern in the network of genes having the same response to environmental changes. We developed our method further by integrating a combination of well established machine learning techniques, which enable the discovery of more complex regulatory patterns. In so doing, we were able to reveal interesting switches that are posted at process bifurcations, in rather good agreement to the expected anaerobic response of hetero-fermentative bacteria.

## Results and discussion

### Extracted discriminative patterns

**Figure 1:**
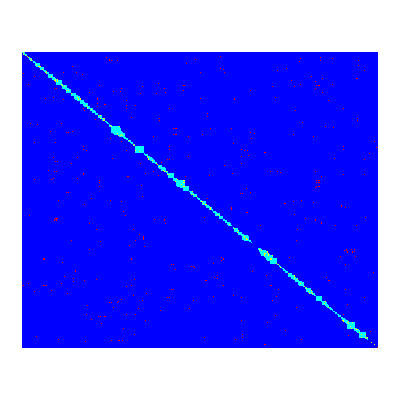
Cluster-matrix. The whole matrix was divided into nine parts. This is a visualisation of the first sector ranging from vertex 1 to 918. True positives and true negatives are coloured in cyan and dark blue, false positives and false negatives in yellow and red, respectively.

**Figure 2:**
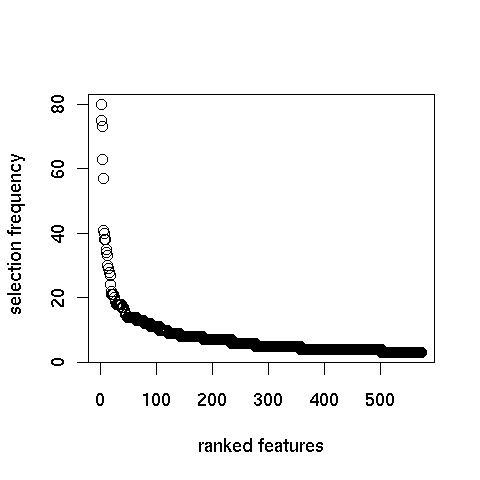
Selection frequencies. Ranked selection frequencies of the features which remained after the recursive feature elimination. The most selected feature was selected 80 times out of 100.

**Figure 3:**
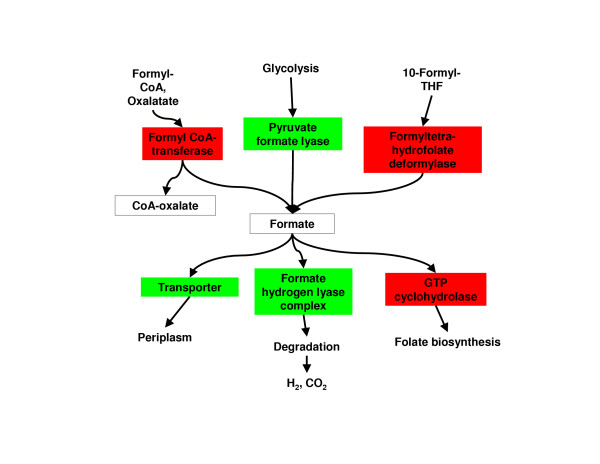
First extracted sub-graph. Green boxes indicate significant up-regulation (p-value ≤ 0.05) under anaerobic conditions. Red boxes indicate significant down-regulation and grey boxes non-significant differential regulation. Red/green frames of grey boxes indicate a non-significant tendency. Glucose is catabolised into pyruvate. Under anaerobic conditions, pyruvate is degraded to formic acid (formate), which is either expelled (via transporters), or further degraded into H_2 _and CO_2_. The reactions for these processes were up-regulated whereas the biosynthesis and degradation of costly compounds were down-regulated (folate and 10-formyl-THF, respectively).

**Figure 4:**
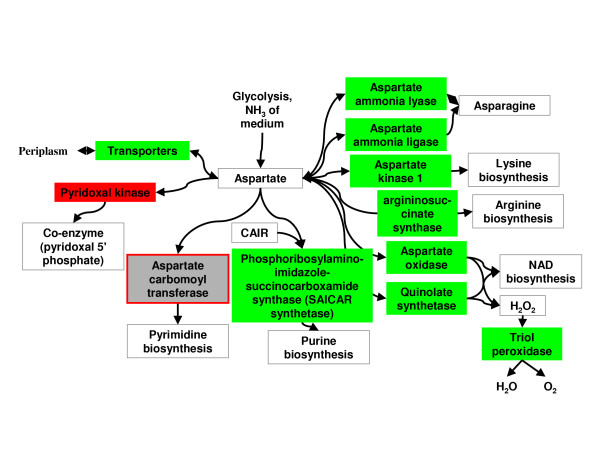
Fourth and tenth extracted sub-graph. It shows the enhanced regulation of some reactions metabolising aspartate in accordance to a higher abundance of aspartate during oxygen deprivation (see text). Aspartate carbamoyl transferase was slightly, but not significantly down-regulated (p-value: 0.16). For box colours see legend of Figure 3.

**Figure 5:**
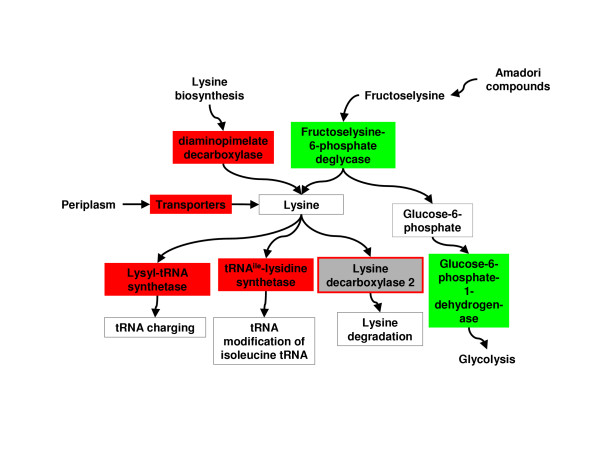
Seventh extracted sub-graph. Lysine biosynthesis, degradation (lysine decarboxylase, p-value: 0.16) and charging and modification of its corresponding tRNAs was down-regulated, while degradation of fructoselysine and its further processing into the glycolysis pathway was up-regulated. For box colours see legend of Figure 3.

**Figure 6:**
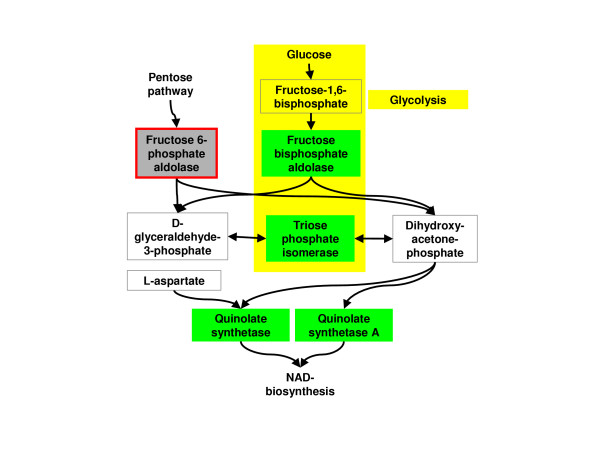
Ninth extracted sub-graph. Interface of the glycolysis (up-regulated, yellow box) and an NAD biosynthesis pathway (see text). For box colours see legend of Figure 3.

The adjacency matrix of the metabolic network was clustered with a variety of penalty parameters (d = 0, -0.05,, -0.9, step = 0.05), obtaining the most suitable clusters with d = -0.1 (Figure 1). This yielded 973 (not necessarily disjoint) clusters of sizes between 2 and 46 reactions. The expression data of all 43 samples (21 aerobic and 22 anaerobic) from the study of Covert et al. [15] were mapped and features for each sample calculated by applying the Haar-wavelet transformations on gene expression patterns of the clusters. We yielded 160,264 features for every sample. After deleting features that consisted of zeros from each sample, 70,912 features remained. A modified t-test was performed [16] to reduce the remaining features and focus the classifier on the most relevant patterns. As a threshold, a false discovery rate of 2e-05 was chosen to further analyse the 9,996 most significant features. With these features, the SVM was trained and tested by a ten-time's ten-fold cross-validation. A recursive feature elimination [17] was applied for each run, yielding 100 lists of the most discriminating features. These features were ranked according to their selection frequency (Figure 2). 8,191 out of 9,996 features were selected at least once. To help us focus on the most relevant features, only features with a significant selection frequency were used (p-value ≤ 0.05, in comparison to a random selection, Bonferroni corrected for multiple testing [18]). This yielded 181 features. Network clusters that contained these features were extracted and are further referred to as "extracted sub-graphs". Extracted sub-graphs were listed in accordance to their selection frequency. All extracted sub-graphs are given in the supplementary material (see Additional file 2, Table S2). In the following, extracted sub-graphs that contained less than six nodes are not considered to focus on larger patterns. Doubles are considered once. The remaining first 10 extracted sub-graphs are listed in Table 1 and are described in detail in the following (Figures 3, 4, 5, 6). Reactions were regarded as up-regulated (green in figures) if the corresponding genes were significantly up-regulated under anaerobic conditions(p-value ≤ 0.05 of a t-test), down-regulated if significantly down-regulated (red in figures), and not significantly differentially regulated otherwise (grey in figures, red/green frames indicate a non-significant tendency). Note, that not all reactions are shown in the figures (especially the non-significantly regulated reactions may not be shown).

The extracted sub-graphs 1, 2, 5 and 6 show the fermentation of formate. In the extracted sub-graph 1 (Figure 3), all edges were due to the metabolite formate, except for the edges coming from dihydroneopterin triphosphate 2'-epimerizase and dihydroneopterin triphosphate pyrophosphohydrolase. Under anaerobic conditions pyruvate formate lyase was up-regulated to process pyruvate into formate (fermentation). To avoid additional production of formate, the other formate producing nodes in the sub-graph were down-regulated. Formate degradation was supported by up-regulated formate hydrogen lyase, which degrades formate into CO_2 _and H_2_. Formate transport into the periplasm was facilitated by the up-regulated expression of the associated transporter gene. Degradation of the quite costly 10-formyl-THF into formate and CoA-transfer for formate production was down-regulated, as formate is abundant enough under anaerobic conditions. Similarily, the GTP cyclohydrolases were down-regulated to limit the biosynthesis of folate.

The extracted sub-graph 3 reveals a basic switching of the leucine transporters. The putative sodium/branched chain amino acid symporter, BrnQ, (see [19,20]) was up-regulated while the ABC-transporter was down-regulated. Both are responsible for leucine uptake. However, BrnQ requires a Na^+ ^gradient whereas the ABC-transporter is dependent on ATP which is limited under anaerobic conditions.

Extracted sub-graphs 4 and 10 (Figure 4) contained reactions involved in anaerobic utilisation of aspartate. This makes sense when taking the support of glucose and ammonium from the medium into account, as in anaerobically grown *E. coli *the glyoxylate cycle is separated into an oxidative and a reductive branch terminating at oxoglutarate and succinyl-CoA. Oxoglutarate and succinyl-CoA have anabolic functions and are required as precursors for glutamate and other syntheses. Aspartate can be produced by aspartate transaminase from oxalacetate using glutamate which can incorporate ammonium from the ammonium rich M9-medium. Indeed, in yeast, it was shown that the aspartate concentration is roughly 100 times higher in the cells under anaerobic conditions (results of Villas-Boas, reported in [21]). The generated aspartate may facilitate the biosynthesis of further amino acids and other important compounds. The genes for the corresponding enzymes were up-regulated in a combined manner. The central role of aspartate was further reinforced by the up-regulation of aspartate transporters. The gene for pyrimidine biosynthesis was slightly but not significantly down-regulated (aspartate transcarbamoylase, p-value: 0.16). Note, that the synthesis of pyrimidine may not be regulated on a transcriptional level. In *E. coli *the committed step for the pyrimidine biosynthesis is aspartate transcarbamoylase, at which ATP and CTP compete for the same site on the regulatory subunit. ATP is activating and CTP inhibiting. As ATP is a purine and CTP a pyrimidine, the ATP/CTP ratio reflects the balance between these types of nucleotides [1]. As energy is limited, the ATP concentration is low under anaerobic conditions, enforcing the inhibiting effect to produce pyrimidine and bringing CTP on an equivalent low level. A quite different regulatory behaviour can be seen regarding purine biosynthesis: SAICAR synthetase is the eighth step in this process. Its required substrate, CAIR, competes with the substrates ATP and aspartate. CAIR binds 200 times more tightly to the free enzyme than to the ternary enzyme [22]. This explains the up-regulation of SAICAR synthetase which we observed, giving CAIR a better chance to bind a free enzyme within the aspartate enriched cytoplasm under anaerobic conditions. Pyridoxal kinase-2 is needed for the biosynthesis of the co-enzyme pyridoxal 5' phosphate. It was down-regulated to save resources, whereas the peroxidases were up-regulated in accordance to safely remove H_2_O_2_.

The seventh extracted sub-graph (Figure 5) shows a general down-regulation of lysine metabolism, such as biosynthesis, degradation, up-take, charging and modification of tRNAs. The sub-graph also reports its interface to the fructoselysine degradation pathway which, interestingly, was up-regulated. Note, that fructoselysine is a degradation product of Amadori compounds (glycolated proteins, see [23]) which degrade poorly under anaerobic conditions [24].

The eighth sub-graph shows elements of the link between energy storing molecules to glycolysis, which was up-regulated under anaerobic conditions. The degradation of malto-dextrin and maltose leads to the release of beta-D-glucose. Beta-D-glucose is converted by glycokinase into beta-D-glucose-phosphate, to be used in glycolysis. The degradation of beta-D-glucose to glucono-delta-lactone was reduced due to the impairment of the electron transfer chain under anaerobic conditions, which is required to further process the resulting ubiquinol. In order to save resources under anaerobic conditions, enzymes participating in the anabolism of galactose and the cata- and anabolism of trehalose were down-regulated. In addition, maltose transporter, maltose-acetyl-transferase and amylomaltase were down-regulated to react upon the exclusive external energy supply by glucose.

The ninth extracted sub-graph (Figure 6) consisted of enzymes at the interface of the glycolysis and an NAD biosynthesis pathway. The higher expression of glycolytic enzymes may indicate an enforced glycolytic turnover of glucose as glycolysis becomes the major energy supply during oxygen deprivation. More interestingly, the glycolysis intermediate dihydroxy acetone phosphate is taken up by up-regulated quinolate synthetases which are the starting point of the NAD biosynthesis. Even though NAD may be more constitutively produced, this makes sense, as it could be shown that quinolate synthetases become inactive when exposed to oxygen [25] and NAD may be primarily produced via the tryptophan biosynthesis pathway under aerobic conditions.

### Comparison to a standard feature extraction method

To compare our findings to a standard feature extraction method, we applied the established feature elimination method by Ruschhaupt et al. [17] to the gene expression levels for the corresponding reactions (without any network information). A ten-time's ten-fold cross-validation was performed on Support Vector Machines, the feature extraction method was applied and the selected features then ranked according to their selection frequency. Table 2 shows the results for the first 40 top ranking reactions. At the top of the table is a formate transporter, pyruvate formate lyase and the formate hydrogen lyase complex, with ranks 1, 2 and 4, respectively. This compares to the first extracted sub-graph (formate metabolism) we found using our method, which may be further extended by formate dehydrogenase (rank 11) and pyruvate formate lyase (ranks 17 and 18). Note that with exception of one reaction of extracted sub-graph 9 (triose phosphate isomerase, rank 21) no other reactions of our extracted sub-graphs could be found by this standard method when considering the first 40 top ranking reactions. This supports our concept of identifying complex expression patterns that may not be found in a common straightforward manner.

### Comparison with the results of the original study

We took the raw gene expression data from Covert and his co-workers [15]. They characterised the regulatory network of *E. coli *with respect to the aerobic – anaerobic shift and compared the gene expression changes of these regulatory genes with the predictions of their former model and their new model. To compare their results with our results, we selected all genes from this regulatory network (Fig. 2 in [15]) and mapped them on the reactions of the proteins they code for. Only genes that had received a corresponding reaction in at least one of our clusters were considered (see Additional file 1, Table S1 in the supplementary material). We could map 126 of 174 genes. From this list, we characterised a gene as "positive" if its corresponding reaction appeared in the extracted sub-graphs (p-value ≤ 0.05) and as "negative" if otherwise. We treated the modelling results of Covert et al. as a 'gold standard' and defined correct predictions of Covert et al. as "true", and incorrect as "false". We yielded a good agreement of our findings with their newer, well elaborated model (precision = 0.78). The precision was much lower, when taking the first model of Covert et al. as the standard (precision = 0.23), supporting the improvement of their modelling method. The recall was reasonable for both models (0.76 and 0.63 for the old and the new model, respectively). Note that, the precision was calculated by dividing the true positives by all positives. Recall was calculated by dividing the true positives by all Covert's correctly predicted genes.

### Discriminative power of the expression data

To test the discriminative power of the expression data, the classification was conducted with the gene expression data alone. Using only the expression data, 42 of 43 samples were correctly classified. Note that we yielded the same classification performance with our method.

### Discussion of the clustering algorithm

Clustering the network consisted of two steps. The computationally more demanding first step computed the optimal cluster matrix for a fixed permutation in linear time, with respect to the number of non-zero entries in the matrix. This exploited the symmetry and could therefore improve the running time by a factor of two. We defined a penalty parameter d to select the clustering stringency. This enabled us to adapt the algorithm to a large variety of network topologies. In the non-simultaneous case the consecutive ones property is regarded for rows only. Christof, Oswald and Reinelt [26] successfully used the results of Tucker [27] and Booth & Lueker [28] to develop a branch-and-cut algorithm for the non-simultaneous case to solve the physical mapping problem [26,29]. Alizadeh et al. [30] and Greenberg et al. [31] used Hamming distance TSP heuristics to solve the underlying consecutive ones problem approximately. Whether the Hamming distance TSP approach works also in the simultaneous case must be tested in the future. Note that optimising *simultaneous *consecutive ones matrices is substantially more difficult than tackling the physical mapping problem. Nevertheless, based on the PQ-Tree-algorithm we were able to implement a fast heuristics and yielded reasonable clustering results. Biological networks contain few nodes with high connectivity, so called "hubs" [32]. These are not easy to group as they may be included in several clusters. On our cluster-matrices we got off-diagonal entries that were not sorted into appropriate clusters (red dots in Figure 1). This problem has to be tackled, for example by testing ensemble methods.

## Conclusion

Our method facilitated the discovery of interesting and complex regulated sub-graphs by testing all possible patterns within the metabolic network and sorting out the patterns with the strongest differences between the conditions. It may suit for a variety of further biological interaction networks, such as signalling networks, e.g. when analysing discriminative regulations in cancers with different prognoses, and/or incorporating interaction data from modern high throughput methods, such as yeast-two-hybrid, chip-on-chip or fluorescence based technologies.

In our case study, we found a strong differential expression pattern of the transcripts coding for formic acid processing enzymes at the interface of the aerobic and anaerobic glucose catabolism: the aerobic catabolism processes pyruvate further on the respiratory glyoxylate cycle, whereas an anaerobic processing uses pyruvate formate lyase to produce formic acid as a fermentative product to be further degraded or excreted. Pyruvate formate lyase may serve as a single switch. However, our study highlighted a concerted regulation reaction on oxygen deprivation. The bacteria adapted to this environmental change not only by degrading pyruvate into formate, but also by reducing formate production from e.g. 10-formyl-THF. Furthermore, formate removal was enhanced by up-regulated genes for formate exocytosis and formate degradation. We revealed an adapted regulation for aspartate processing enzymes. Interestingly, coming from aspartate, the starting point for purine biosynthesis was up-regulated, whereas that of pyrimidine biosynthesis was not. Searching for an explanation, we identified recent articles and textbook entries which provide plausible reasoning for this situation (see Results and Discussion). Further switches were revealed, as e.g. the activation of the NAD biosynthesis pathway under oxygen deprivation.

Hence, we elucidated some interesting and relevant sub-graphs of the metabolic network that showed necessary changes during the aerobic – anaerobic shift. But note, that such findings may not represent the entire regulatory change during such a shift of the metabolic network.

## Methods

### Establishing the network

**Figure 7:**
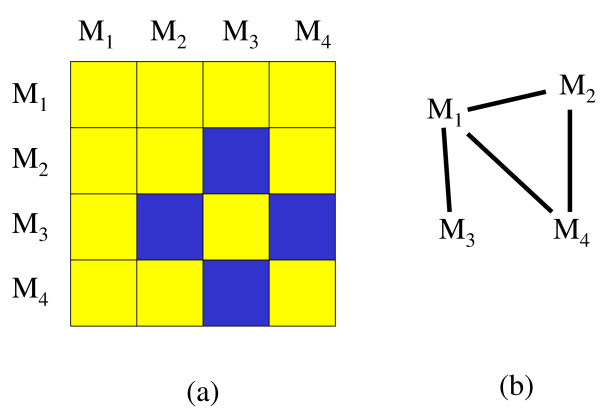
Simple example of a cluster sub-matrix. The cluster is represented by its matrix (a) and its corresponding sub-graph (b). "1" entries are designated as yellow boxes, "0" entries as blue boxes. Note, that the main diagonal entries are set to "1".

Metabolic reactions were extracted from the EcoCyc database (Version 9, [33]). A graph was established by defining neighbours of metabolites. Two metabolites were neighbours if and only if an enzymatic reaction existed that needed one of the metabolites as input (needed substrate) and produced the other as output (product). Note, that in this representation, enzymes are edges and metabolites the nodes. This network was clustered to group enzymes into parts of the network with their major connections (the clustering algorithm is described below, see section "The clustering method"). The clustering algorithm produced a symmetrical sub-matrix of the cluster matrix for each cluster, whose rows and columns were the metabolites. The matrix contained a "1" entry at position (i, j) if an enzyme existed that combined metabolites of row i and column j. Otherwise a "0" entry was set (Figure 7).

### Mapping gene expression data onto the cluster-matrices

For our case study, we collected raw intensity values of gene expression data from the work of Covert et al. [15] which we downloaded from the ASAP database [34]. Covert et al. determined mRNA levels of all open reading frames by hybridisations on Affymetrix oligo microarrays. We normalised them with an established variance normalisation method [35] and selected the data for 43 hybridisations of the following samples: strain K-12 MG 1655, wild-type, Δ*arcA, ΔappY, Δfnr, ΔoxyR, ΔsoxS *single mutants and the Δ*arcAΔfnr *double mutant (for generation of the knockouts and growth conditions, see [36,37], respectively). The mutated genes are key transcriptional regulators of the oxygen response [15]. They effect a major portion of all genes in *E. coli *and therefore supported a variance stimulation of the respiratory and fermentative control of the investigated strain. All gene expression experiments were done in triplicate under aerobic and anaerobic conditions, respectively, except for anaerobic wild-type which was repeated four times. The gene expression data of each data-set was mapped onto the corresponding reactions of the transcribed proteins. Mean values were taken if a reaction was catalysed by a complex of proteins. The expression data of all samples was mapped onto each cluster-matrix, yielding 43 different patterns for each cluster.

### Pattern discovery: defining the features with the Haar wavelet transform

We wanted to calculate a value for every possible expression pattern of neighbouring genes and groups of genes within a cluster that may show essential differences between samples of different conditions. Therefore, we performed a Haar-wavelet transform for each cluster-matrix. The wavelet transformed expression values served as features for the classifier (classification method, see next section). This allowed the identification of regions with a varying pattern between aerobic and anaerobic conditions. The wavelet-transformation is described in the following. Each cluster-matrix was divided into 2 × 2 pixeled disjoint sub-sections (e.g. a cluster matrix of size 8 × 8 was divided into 16 sub-sections). Clusters with non-fitting sizes (e.g. 3 × 3, 5 × 5,) were extended with rows and columns of zeros to yield matrices that could be divided into 2 × 2 pixeled sub-sections. For each sub-section, all combinations of row-wise and column-wise mean and differences, respectively, were calculated. This yielded 4 combined values for each 2 × 2 pixeled sub-section: 1st: mean of the mean of the upper and mean of the lower row, 2nd: difference of the mean of the upper and the mean of the lower row, 3rd: mean of the difference of the upper and the difference of the lower row, and, 4th: difference of the difference of the upper and the difference of the lower row. All four combined values for each 2 × 2 pixeled sub-section were stored and applied as features for the classifier. This was done for all sub-sections of the matrix. All 1st combined values (mean of means) were taken for a new matrix and were again grouped into 2 × 2 fractions that were combined in the same manner, yielding again 4 new features for every fraction. This procedure was repeated until no further grouping was possible. Such a "Haar" wavelet transform can be regarded as a low pass filter when calculating the mean, and a high pass filter when calculating the difference between neighbouring value pairs. The transform applied a filter in horizontal and subsequently in vertical direction. The procedure consisted of repeatedly applying high and low pass filters on the image. Therefore, either high frequency or low frequency portions of the signal were calculated and stored, until the maximal possible compositions were obtained. This procedure was carried out for all clusters of every sample and the results of the transforms were stored as the corresponding features for every sample.

### Extracting essential features and their sub-graphs with the classifier

The SAM method [16] as a modified t-test was performed to rank the features according to their p-values. Higher ranking features (low p-values) were selected focusing the classifier on the most relevant patterns (9,996 out of 70,912). For classification, we applied the Support Vector Machine implementation as provided by the R MCRestimate package [17]. To receive a suitable feature extraction result, a 10-fold cross validation was performed and repeated 10 times with different splittings of the data, respectively. A linear kernel was applied for the feature extraction as described elsewhere [17]. Parameter optimisation was performed for the regularisation term that defined the costs for false classifications (9 steps, range: 2^n^, n = -4, -2,, 8, 10). This optimisation was realised by an internal three-fold cross validation during every iteration. To determine the most relevant features, a recursive feature elimination [17] was applied during the parameter optimisation procedure. This yielded a set of discriminating features for every run. These features were ranked due to their selection frequency of all 100 runs. Note, that high-ranking features yielded the corresponding sub-graphs (cluster of the cluster matrix) of the reaction network that contained well discriminating patterns of the expression data. We defined a cut-off criterion for selecting only substantial features by comparing the selection frequency of each feature with random selections. We assumed a binomial distribution, neglecting the cases that the same feature may have been chosen twice in one run. The overall number of drawings was the sum of all selections (8,191 selections). The probability to draw the respective feature was the reciprocal value of the number of all features (1/9,996). The number of drawings for the respective feature was its selection frequency. As we calculated this for every feature, the resulting p-values where corrected for multiple testing by multiplying them with the number of all features (Bonferroni correction [18]).

### The clustering method

The metabolic network was represented by an adjacency matrix: An entry at row i and column j was set to "1" if there existed a common reaction downstream of metabolite i and upstream of metabolite j or *vice versa*. Note, that for the pattern discovery method described above, this entry was the corresponding gene expression value of the reaction. The adjacency matrix consisted of 2,754 rows and columns and 2 × 17,233 non-zero entries (entries (i, j) and (j, i) equaled). Hence, non-zero entries were rare (≈ 0.45%). The matrix was transformed to obtain regions enriched with non-zero elements by the following clustering method.

**Figure 8:**
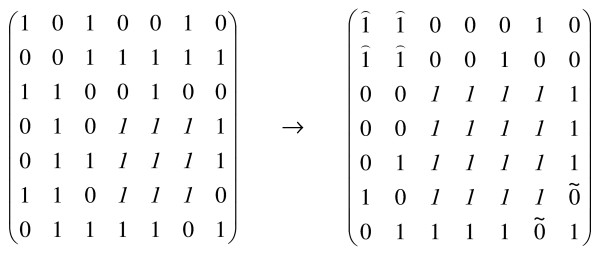
Example of an adjacency matrix for 7 nodes. Permuting rows and columns 2 and 3 yields a better clustering with two clusters, indicated by hats and italic digits, respectively. Flipping "0" entries on the bottom right of the matrix (marked with tildes) further extends the cluster with italic digits.

Given the metabolic network as graph G(V,E) with node set V (metabolites) and edge set E (reactions), our goal was to identify clusters of G where each cluster was given by the node set of a highly connected sub-graph. Note, that we did not require the clusters to be mutually disjoint. The clustering algorithm based on the so-called *weighted simultaneous consecutive ones problem *.Clusters were represented by symmetric 0/1-matrices. Rows and columns of the matrix corresponded to the nodes of G. A "1" entry in position (i, j) indicated that there was at least one cluster containing both node i and node j. A "0" entry indicated that there was no cluster containing both nodes. The diagonal entries were fixed to "1" (Figure 7). We call such a matrix *cluster-matrix*. Using a suitable permutation to rearrange both the rows and the columns of the cluster-matrix, one can obtain a matrix with consecutive "1" entries in every row as well as in every column. Note, that a matrix has the *simultaneous consecutive ones property (SCO) *,if such a rearrangement of rows and columns is possible. A characterisation of such matrices was given by Tucker [27] and there is a linear time algorithm for checking this property using the PQ-Tree algorithm introduced by Booth and Lueker [28]. This algorithm outputs a data structure from which all node permutations that establish the SCO property can be generated. The basic task was to convert the adjacency matrix (A) of G into a cluster-matrix X containing only "1" in the clusters. Altering an entry of the adjacency matrix ("0" to "1" flip) was allowed though penalised. The main part of this process was to search for simultaneous permutations (rows and columns) that led to a minimum number of "0" to "1" flips. Figure 8 shows an example. We parameterised the objective function using a negative parameter d for adjusting the ratio between rewarding an edge inside a cluster and penalising a "0" to "1" flip, i.e. for placing two nodes that were not connected inside the same cluster. Formally, given a symmetric adjacency matrix A^n x n ^with a_ij _∈ {0, 1} and a_ii _= 1 ∀ i, j = 1...n, we wanted to maximise the objective function

cX:=∑ijcijxij,     (1)
 MathType@MTEF@5@5@+=feaafiart1ev1aaatCvAUfKttLearuWrP9MDH5MBPbIqV92AaeXatLxBI9gBaebbnrfifHhDYfgasaacH8akY=wiFfYdH8Gipec8Eeeu0xXdbba9frFj0=OqFfea0dXdd9vqai=hGuQ8kuc9pgc9s8qqaq=dirpe0xb9q8qiLsFr0=vr0=vr0dc8meaabaqaciaacaGaaeqabaqabeGadaaakeaacqWGJbWydaahaaWcbeqaaiabdIfaybaakiabcQda6iabg2da9maaqababaGaem4yam2aaSbaaSqaaiabdMgaPjabdQgaQbqabaGccqWG4baEdaWgaaWcbaGaemyAaKMaemOAaOgabeaaaeaacqWGPbqAcqWGQbGAaeqaniabggHiLdGccqGGSaalcaWLjaGaaCzcamaabmaabaGaeGymaedacaGLOaGaayzkaaaaaa@433E@

where the coefficients c_ij _were defined by

cij:={1ifaij=1delse.     (2)
 MathType@MTEF@5@5@+=feaafiart1ev1aaatCvAUfKttLearuWrP9MDH5MBPbIqV92AaeXatLxBI9gBaebbnrfifHhDYfgasaacH8akY=wiFfYdH8Gipec8Eeeu0xXdbba9frFj0=OqFfea0dXdd9vqai=hGuQ8kuc9pgc9s8qqaq=dirpe0xb9q8qiLsFr0=vr0=vr0dc8meaabaqaciaacaGaaeqabaqabeGadaaakeaacqWGJbWydaWgaaWcbaGaemyAaKMaemOAaOgabeaakiabcQda6iabg2da9maaceaabaqbaeaabiWaaaqaaiabigdaXaqaaiabdMgaPjabdAgaMbqaaiabdggaHnaaBaaaleaacqWGPbqAcqWGQbGAaeqaaOGaeyypa0JaeGymaedabaGaemizaqgabaGaemyzauMaemiBaWMaem4CamNaemyzaugabaaaaaGaay5EaaGaeiOla4IaaCzcaiaaxMaadaqadaqaaiabikdaYaGaayjkaiaawMcaaaaa@4950@

X = (x_ij_) denoted the cluster-matrix of A to be optimised. As a starting point, X equaled A with all diagonal entries fixed to "1". The goal was to transform X by simultaneous column- and row-permutations yielding a matrix that consisted of quadratic sub-matrices with "1" entries and "0" entries else.

Note that, as we allowed missing connections inside a cluster, non-connected nodes could still enter the same cluster. The objective function was penalised by a negative value d for every c_ij _inside a cluster of X that was a "0" entry in A. This avoided clusters with too many non-existing connections in it. If the nodes i and j were not connected by an edge (i.e. a_ij _= 0) but assigned to the same cluster (i.e. x_ij _= 1), the negative parameter d reduced the objective function value c^X^. Note that, as the number of inequalities for describing the SCO grows exponentially with the input elements, it got computationally too hard to solve the integer program exactly for this study. Therefore, we designed a fast heuristics based on algorithms for the consecutive ones problem. Having as an input the adjacency matrix A of our graph G to obtain the clustering matrix X^opt^, the heuristics worked as follows:

1 Fix all diagonal entries a_ii _of A to 1

2 Set c^Xopt ^= - ∞

3 Define X^opt ^to have 1-entries only in the main diagonal

4 Compute the coefficient matrix C = (c_ij_) defined by c_ij_: = 1 if a_ij _= 1 and c_ij_: = d if a_ij _= 0

5 FOR i = 1,..., k DO

5.1 Initialise a random permutation π_i _of X

5.2 FOR j = 0, ..., l DO

5.2.1 Use dynamic programming to compute an optimal cluster matrix X for this fixed permutation π_i_

5.2.2 IF c^X ^> c^Xopt^

5.2.2.1 Update X^opt ^and c^Xopt^

5.2.3 Use the PQ-Tree-Algorithm to select randomly a permutation from all permutations that keep the found clusters together

6 Try to improve X^opt ^by m iterations of loop 5.2

The choice of the loop parameters depended on the size of the input and was chosen as k = 250, l = 10 and m = 100. The two loops were independent of each other. The outer loop 5 restarted the main part of the heuristics (loop 5.2) for several different start permutations, always saving the best result found.

## List of abbreviations

SAICAR synthetase: phosphoribosylaminoimidazolesuccinocarboxamide synthetase;

CAIR: 5-aminoimidazole-4-carboxyribonucleotide.

## Authors' contributions

RK and GS conceptualised and designed the method and analysed the data. HS, MO, SS, GR conceptualised the clustering method and generated the clustering data. RK and MZ analysed and interpreted the data on its biological content. RE initiated the co-operation between RK, GS, MZ, RE and MO, HS, SS, GR. The manuscript was written by RK, GS, MO and HS. RE revised it critically. All authors read and approved the final manuscript.

## Supplementary Material

Additional File 2 All extracted sub-graphs Click here for file

Additional File 1 Comparison of our results with the model predictions of Covert et al. [15] Click here for file

## Figures and Tables

**Table 1:** Extracted sub-graphs. EcoCyc-ids of the reactions, their corresponding enzyme annotations, their observed regulation due to a changing from aerobic to anaerobic conditions and the confidence value for this observation. Listed are the first 10 extracted sub-graphs.

**id**	**enzymes**	**regulation**	**p-value**
***1st extracted sub-graph (formate metabolism)***			
FHLMULTI-RXN	formate hydrogenlyase complex	1	3.8e-16
FORMYLTHFDEFORMYL-RXN	formyltetrahydrofolate deformylase	-1	0.0015
GTP-CYCLOHYDRO-I-RXN	GTP cyclohydrolase I	-1	0.00088
GTP-CYCLOHYDRO-II-RXN	GTP cyclohydrolase II	(1)	0.57
H2NEOPTERINP3PYROPHOSPHOHYDRO-RXN	dihydroneopterin triphosphate pyrophosphohydrolase	(0)	1
H2NTPEPIM-RXN	dihydroneopterin triphosphate 2'-epimerase	-1	1.024e-05
KETOBUTFORMLY-RXN	2-ketobutyrate formate-lyase	(-1)	0.20
PYRUVFORMLY-RXN	pyruvate formate-lyase	1	2.2e-18
RXN0-1382	formyl-CoA transferase	-1	0.023
TRANS-RXN-1	transporter	1	4.5e-20
***2nd extracted sub-graph (formate metabolism)***			
FHLMULTI-RXN	formate hydrogenlyase complex	1	3.8e-16
FORMYLTHFDEFORMYL-RXN	formyltetrahydrofolate deformylase	-1	0.0015
GTP-CYCLOHYDRO-I-RXN	GTP cyclohydrolase I	-1	0.00088
GTP-CYCLOHYDRO-II-RXN	GTP cyclohydrolase II	(1)	0.57
KETOBUTFORMLY-RXN	2-ketobutyrate formate-lyase	(-1)	0.20
PYRUVFORMLY-RXN	pyruvate formate-lyase	1	2.2e-18
RXN0-443		(0)	1
TRANS-RXN-1	transporter	1	4.5e-20
***3rd extracted sub-graph (leucine)***			
ABC-35-RXN	Transporters	-1	4.1e-09
BRANCHED-CHAINAMINOTRANSFERLEU-RXN	branched chain amino acid aminotransferase	1	0.080
LEUCINE – TRNA-LIGASE-RXN	leucyl-tRNA synthetase	(1)	0.13
LEUCYLTRANSFERASE-RXN	leucyl phenylalanyl-tRNA-protein transferase	1	0.011
RXN0-261		(0)	1
TRANS-RXN-126B	transporter	1	6.2e-10
***4th extracted sub-graph (aspartate metabolism)***			
2-METHYLCITRATE-SYNTHASE-RXN	methylcitrate synthase	(1)	0.32
ARGSUCCINSYN-RXN	argininosuccinate synthase	1	0.027
ASNSYNA-RXN	aspartate-ammonia ligase	1	0.0032
ASPARTASE-RXN	aspartate ammonia-lyase	1	1.6e-06
ASPARTATE – TRNA-LIGASE-RXN	aspartyl-tRNA synthetase	(1)	0.61
ASPARTATEKIN-RXN	aspartate kinase I	1	0.0082
ASPCARBTRANS-RXN	aspartate carbamoyltransferase	(-1)	0.16
ASPDECARBOX-RXN	aspartate-1-decarboxylase	(1)	0.23
L-ASPARTATE-OXID-RXN	L-aspartate oxidase	1	0.00033
PEPCARBOXYKIN-RXN	phosphoenolpyruvate carboxykinase (ATP)	(1)	0.58
PEROXID-RXN	thiol peroxidase	(1)	0.20
PYRIDOXKIN-RXN	pyridoxal kinase 2	(-1)	0.045
PYROXALTRANSAM-RXN	pyridoxamine-oxaloacetate transaminase	(0)	1
QUINOLINATE-SYNTHE-MULTI-RXN	quinolinate synthetase	1	0.00051
RXN0-267	thiol peroxidase 2	-1	8.1e-08
SAICARSYN-RXN	phosphoribosylaminoimidazole- succinocarboxamide synthase	1	9.0e-07
TRANS-RXN-106A	transporter	1	0.015
TRANS-RXN-122A	transporter	1	2.2e-06
***5th extracted sub-graph (formate metabolism)***			
FHLMULTI-RXN	formate hydrogenlyase complex	1	3.8e-16
FORMYLTHFDEFORMYL-RXN	formyltetrahydrofolate deformylase	-1	0.0015
GTP-CYCLOHYDRO-I-RXN	GTP cyclohydrolase I	-1	0.00088
GTP-CYCLOHYDRO-II-RXN	GTP cyclohydrolase II	(1)	0.57
H2NEOPTERINP3PYROPHOSPHOHYDRO-RXN	dihydroneopterin triphosphate pyrophosphohydrolase	(0)	1
H2NTPEPIM-RXN	dihydroneopterin triphosphate 2'-epimerase	-1	1.0e-05
KETOBUTFORMLY-RXN	2-ketobutyrate formate-lyase	(-1)	0.2
PYRUVFORMLY-RXN	pyruvate formate-lyase	1	2.2e-18
RXN0-443		(0)	1
TRANS-RXN-1	transporter	1	4.5e-20
***6th extracted sub-graph (formate metabolism)***			
3.5.1.88-RXN	dihydroxyacetone kinase	-1	0.059
FORMYLTHFDEFORMYL-RXN	formyltetrahydrofolate deformylase	-1	0.0015
GARTRANSFORMYL2-RXN	GAR transformylase 2	(-1)	0.41
GTP-CYCLOHYDRO-I-RXN	GTP cyclohydrolase I	-1	0.00088
METHENYLTHFCYCLOHYDRO-RXN	methenyltetrahydrofolate cyclohydrolase	1	0.0052
PYRUVFORMLY-RXN	pyruvate formate-lyase	1	2.2e-18
RXN0-1382	formyl-CoA transferase	-1	0.023
RXN0-443		(0)	1
TRANS-RXN-1	transporter	1	4.5e-20
***7th extracted sub-graph (lysine)***			
DIAMINOPIMDECARB-RXN	diaminopimelate decarboxylase	-1	0.0012
GLU6PDEHYDROG-RXN	glucose 6-phosphate-1-dehydrogenase	1	0.027
LYSDECARBOX-RXN	lysine decarboxylase 2	(-1)	0.14
LYSINE – TRNA-LIGASE-RXN	lysyl tRNA synthetase	-1	0.0060
RXN0-1961	tRNA-Ile-lysidine synthetase	-1	0.015
RXN0-963	fructoselysine 6-phosphate deglycase	1	0.0059
TRANS-RXN-58	Transporter	-1	0.049
TRANS-RXN-68	Transporter	(0)	0.81
***8th extracted sub-graph (Glycolysis)***			
6-PHOSPHO-BETA-GLUCOSIDASE-RXN	phenylacetate-CoA ligase	1	2.9e-06
AMYLOMALT-RXN	amylomaltase	(-1)	0.34
GALACTURIDYLYLTRANS-RXN	UDP-glucose-hexose-1-phosphate uridylyltransferase	-1	1.1e-06
GLUCDEHYDROG-RXN	glucose dehydrogenase (pyrroloquinoline-quinone)	-1	5.3e-05
GLUCOKIN-RXN	glucokinase	1	1.4e-07
GLUCOSE-1-PHOSPHAT-RXN	glucose-1-phosphatase	(0)	0.78
MALTACETYLTRAN-RXN	maltose acetyltransferase	(-1)	0.43
MALTDEG-RXN	maltose degrading enzyme	(-1)	0.34
MALTODEG-RXN	maltose degrading enzyme 2	(-1)	0.34
MALTODEXGLUCOSID-RXN	maltodextrin glucosidase	(1)	0.26
PGLUCISOM-RXN	phosphoglucose isomerase	1	3.3e-07
RXN0-2543	flavorubredoxin reductase	(-1)	0.13
TRE6PHYDRO-RXN	trehalose-6-phosphate hydrolase	(-1)	0.097
TREHALOSE6PSYN-RXN	trehalose-6-phosphate synthase	(0)	0.87
***9th extracted sub-graph (Glycolysis/NAD biosynthesis)***			
QUINOLINATE-SYNTHA-RXN	quinolinate synthetase A	1	0.0016
QUINOLINATE-SYNTHE-MULTI-RXN	quinolinate synthetase	1	0.00051
RHAMNULPALDOL-RXN	rhamnulose-1-phosphate aldolase	(-1)	0.25
RXN0-313	fructose 6-phosphate aldolase 1	(-1)	0.51
TAGAALDOL-RXN	tagatose-16-bisphosphate aldolase 1	(1)	0.60
TRIOSEPISOMERIZATION-RXN	triose phosphate isomerase	1	1.9e-12
***10th extracted sub-graph (aspartate metabolism)***			
2-METHYLCITRATE-SYNTHASE-RXN	methylcitrate synthase	(1)	0.32
ARGSUCCINSYN-RXN	argininosuccinate synthase	1	0.027
ASNSYNA-RXN	aspartate-ammonia ligase	1	0.0032
ASPARTASE-RXN	aspartate ammonia-lyase	1	1.6e-06
ASPARTATE – TRNA-LIGASE-RXN	aspartyl-tRNA synthetase	(1)	0.61
ASPARTATEKIN-RXN	aspartate kinase I	1	0.0082
ASPCARBTRANS-RXN	aspartate carbamoyltransferase	(-1)	0.16
ASPDECARBOX-RXN	aspartate-1-decarboxylase	(1)	0.23
L-ASPARTATE-OXID-RXN	L-aspartate oxidase	1	0.00033
PEPCARBOXYKIN-RXN	phosphoenolpyruvate carboxykinase (ATP)	(1)	0.57
PEROXID-RXN	thiol peroxidase	(1)	0.20
PYRIDOXKIN-RXN	pyridoxal kinase 2	-1	0.045
PYROXALTRANSAM-RXN	pyridoxamine-oxaloacetate transaminase	(0)	1
QUINOLINATE-SYNTHE-MULTI-RXN	quinolinate synthetase	1	0.00051
RXN0-267	thiol peroxidase 2	-1	8.1e-08
SAICARSYN-RXN	phosphoribosylaminoimidazole- succinocarboxamide synthase	1	9.0e-07
TRANS-RXN-122A	transporter	1	2.2e-06

**Table 2:** The 40 first ranking reactions when applying the feature extraction directly without any network information.

Rank	reaction id (EcoCyc)	corresponding enzyme	regulation	p-value	involved in extracted sub-graph
1	TRANS-RXN-1	transporter	1	4.5e-20	1,2,5,6
2	PYRUVFORMLY-RXN	pyruvate formate-lyase	1	2.2e-18	1,2,5,6
3	GCVT-RXN	aminomethyltransferase	-1	2.8e-18	none
4	FHLMULTI-RXN	formate hydrogenlyase complex	1	3.8e-16	1,2,5
5	2PGADEHYDRAT-RXN	enolase	1	5.1e-15	none
6	GCVP-RXN	glycine dehydrogenase (decarboxylating)	-1	6.3e-15	none
7	3-CH3-2-OXOBUTANOATE-OH- CH3-XFER-RXN	3-methyl-2-oxobutanoate hydroxymethyltransferase	1	7.1e-15	none
8	PEPDEPHOS-RXN	pyruvate kinase I	1	9.1e-15	none
9	PFLDEACTIV-RXN	PFL-deactivase	1	3.0e-14	none
10	ACETALD-DEHYDROG-RXN	acetaldehyde dehydrogenase	1	3.0e-14	none
11	FORMATEDEHYDROG-RXN	formate dehydrogenase	1	3.7e-14	none
12	GCVMULTI-RXN	gcv system	-1	7.7e-14	none
13	NACMURLALAAMI-RXN	N-acetylmuramyl-L-alanine amidase	1	8.6e-14	none
14	R601-RXN	fumarate reductase	1	1.1e-13	none
15	MANNONDEHYDRAT-RXN	mannonate dehydratase	-1	2.0e-13	none
16	GLUTDEHYD-RXN	glutamate dehydrogenase (NADP+)	1	2.8e-13	none
17	1.97.1.4-A-RXN	pyruvate formate-lyase activating enzyme	1	4.8e-13	none
18	TDCEACT1-RXN	pyruvate formate-lyase activating enzyme	1	4.8e-13	none
19	GLUTRNAREDUCT-RXN	glutamyl-tRNA reductase	1	1.4e-12	none
20	HISTAMINOTRANS-RXN	histidine-phosphate aminotransferase	1	1.7e-12	none
21	TRIOSEPISOMERIZATION-RXN	triose phosphate isomerase	1	1.9e-12	9
22	MANNPISOM-RXN	mannose-6-phosphate isomerase	1	4.5e-12	none
23	NAD-KIN-RXN	putative NAD+ kinase	1	4.7e-12	none
24	6PFRUCTPHOS-RXN	6-phosphofructokinase-1	1	4.7e-12	none
25	4OH2OXOGLUTARALDOL-RXN	2-keto-4-hydroxyglutarate aldolase	1	6.0e-12	none
26	KDPGALDOL-RXN	2-keto-3-deoxy-6-phosphogluconate aldolase	1	6.0e-12	none
27	OXALODECARB-RXN	oxaloacetate decarboxylase	1	6.0e-12	none
28	ABC-26-RXN	tranport	-1	8.2e-12	none
29	RXN0-2181	asparaginase III	-1	1.4e-11	none
30	RXN0-1682	asparaginase III	-1	1.4e-11	none
31	RXN0-307	NADH oxidoreductase	1	1.8e-11	none
32	RXN0-1565	tRNA (Gm18) 2'-O-methyltransferase	-1	2.5e-11	none
33	TRANS-RXN-242A	Transporter	1	3.0e-11	none
34	PHOSICITDEHASE-RXN	isocitrate dehydrogenase kinase	-1	3.1e-11	none
35	DEPHOSICITDEHASE-RXN	isocitrate dehydrogenase phosphatase	-1	3.1e-11	none
