# Supplementary material for: Discovering functional gene expression patterns in the metabolic network of Escherichia coli with wavelets transforms
Source: BMC Bioinformatics. 2006 Mar 8;7:119. doi: 10.1186/1471-2105-7-119 (PMC1434775; doi:10.1186/1471-2105-7-119)
Supplement: Additional File 1 — Comparison of our results with the model predictions of Covert et al. [15] [file 1471-2105-7-119-S1.doc]

**Table S1**

Comparison of our results with the model predictions of Covert et al (Nature, 429, 94, 2004). Columns "pred 1" and "pred 2" show the prediction performance of Covert et al for their older and newer model, respectively (1, 2: correct prediction, 3-5: wrong prediction). Our results are shown in the last column, i.e. the ranked sub-graph in which the corresponding reaction occurred first. Sub-graphs < 182 were classified as "positive", and as "negative" else.

|  | Locus | Gene | pred 1 | pred 2 | reactions | sub-graph |
| --- | --- | --- | --- | --- | --- | --- |
| 1 | b0828 | ybiK | 5 | 5 | ASPARAGHYD-RXN, | 21 |
| 2 | b1261 | trpB | 5 | 5 | RXN0-2382,TRYPSYN-RXN, | 83 |
| 3 | b2021 | hisC | 5 | 5 | HISTAMINOTRANS-RXN, | 1443 |
| 4 | b2478 | dapA | 5 | 5 | DIHYDRODIPICSYN-RXN, | 158 |
| 5 | b3769 | ilvM | 5 | 1 | ACETOLACTSYN-RXN,ACETOOHBUTSYN-RXN, | 358 |
| 6 | b3770 | ilvE | 5 | 5 | BRANCHED-CHAINAMINOTRANSFERILEU-RXN,BRANCHED-CHAINAMINOTRANSFERLEU-RXN,BRANCHED-CHAINAMINOTRANSFERVAL-RXN, | 4 |
| 7 | b3771 | ilvD | 5 | 5 | DIHYDROXYISOVALDEHYDRAT-RXN,DIHYDROXYMETVALDEHYDRAT-RXN, | 6165 |
| 8 | b3957 | argE | 5 | 5 | ACETYLORNDEACET-RXN, | 44 |
| 9 | b0068 | sfuA | 5 | 1 | ABC-32-RXN, | 2696 |
| 10 | b0133 | panC | 5 | 5 | PANTOATE-BETA-ALANINE-LIG-RXN, | 358 |
| 11 | b0595 | entB | 5 | 3 | ENTG-RXN,ENTMULTI-RXN,ISOCHORMAT-RXN, | 101 |
| 12 | b0776 | bioF | 5 | 1 | 7KAPSYN-RXN, | 440 |
| 13 | b1210 | hemA | 5 | 5 | GLUTRNAREDUCT-RXN, | 1196 |
| 14 | b1991 | cobT | 5 | 1 | DMBPPRIBOSYLTRANS-RXN, | 1135 |
| 15 | b1993 | cobU | 5 | 1 | COBINAMIDEKIN-RXN,COBINPGUANYLYLTRANS-RXN, | 3701 |
| 16 | b2153 | folE | 5 | 5 | GTP-CYCLOHYDRO-I-RXN, | 1 |
| 17 | b3041 | ribB | 5 | 5 | DIOHBUTANONEPSYN-RXN, | 453 |
| 18 | b3368 | cysG | 1 | 1 | DIMETHUROPORDEHYDROG-RXN,SIROHEME-FERROCHELAT-RXN,UROPORIIIMETHYLTRANSA-RXN, | 360 |
| 19 | b3805 | hemC | 5 | 1 | OHMETHYLBILANESYN-RXN, | 2724 |
| 20 | b3990 | thiH | 5 | 1 | THIAZOLSYN2-RXN, | 548 |
| 21 | b3993 | thiE | 5 | 1 | THI-P-SYN-RXN, | 22 |
| 22 | b3994 | thiC | 5 | 1 | PYRIMSYN1-RXN, | 664 |
| 23 | b0904 | focA | 1 | 1 | TRANS-RXN-1, | 1 |
| 24 | b1613 | manA | 5 | 5 | MANNPISOM-RXN, | 235 |
| 25 | b2297 | pta | 5 | 5 | PHOSACETYLTRANS-RXN,PTAALT-RXN, | 309 |
| 26 | b0114 | aceE | 5 | 1 | RXN0-1134,PYRUVATEDECARB-RXN,PYRUVDEH-RXN, | 759 |
| 27 | b0116 | lpdA | 5 | 1 | 2OXOGLUTARATEDEH-RXN,DIHYDLIPOXN-RXN,RXN0-1131,RXN0-1132,RXN0-1142,GCVMULTI-RXN,PYRUVDEH-RXN, | 67 |
| 28 | b0118 | acnB | 5 | 1 | ACONITATEDEHYDR-RXN,ACONITATEHYDR-RXN,RXN0-311, | 159 |
| 29 | b0429 | cyoD | 1 | 1 | CYT-UBIQUINOL-OXID-RXN, | 112 |
| 30 | b0430 | cyoC | 1 | 1 | CYT-UBIQUINOL-OXID-RXN, | 112 |
| 31 | b0431 | cyoB | 1 | 1 | CYT-UBIQUINOL-OXID-RXN, | 112 |
| 32 | b0432 | cyoA | 1 | 1 | CYT-UBIQUINOL-OXID-RXN, | 112 |
| 33 | b0723 | sdhA | 5 | 1 | SUCC-FUM-OXRED-A-RXN,|SUCCINATE-DEHYDROGENASE-(UBIQUINONE)-RXN|, | 158 |
| 34 | b0726 | sucA | 5 | 1 | 2OXOGLUTARATEDEH-RXN, | 274 |
| 35 | b0727 | sucB | 5 | 1 | 2OXOGLUTARATEDEH-RXN, | 274 |
| 36 | b0728 | sucC | 5 | 5 | SUCCCOASYN-RXN, | 274 |
| 37 | b0729 | sucD | 5 | 5 | SUCCCOASYN-RXN, | 274 |
| 38 | b0733 | cydA | 5 | 1 | CYT-UBIQUINOL-OXID-RXN, | 112 |
| 39 | b0734 | cydB | 5 | 1 | CYT-UBIQUINOL-OXID-RXN, | 112 |
| 40 | b0755 | gpmA | 5 | 1 | 3PGAREARR-RXN, | 2865 |
| 41 | b0896 | dmsC | 4 | 2 | DIMESULFREDUCT-RXN, | 52 |
| 42 | b0902 | pflA | 1 | 1 | TDCEACT1-RXN,1.97.1.4-A-RXN, | 44 |
| 43 | b0903 | pflB | 1 | 1 | PYRUVFORMLY-RXN,KETOBUTFORMLY-RXN, | 1 |
| 44 | b0974 | hyaC | 1 | 1 | HYDROG-RXN, | 235 |
| 45 | b1136 | icdA | 5 | 5 | ISOCITDEH-RXN, | 589 |
| 46 | b1241 | adhE | 5 | 1 | ACETALD-DEHYDROG-RXN,ALCOHOL-DEHYDROG-GENERIC-RXN,ALCOHOL-DEHYDROG-RXN,PFLDEACTIV-RXN, | 247 |
| 47 | b1276 | acnA | 5 | 5 | ACONITATEDEHYDR-RXN,ACONITATEHYDR-RXN, | 159 |
| 48 | b1415 | aldA | 5 | 5 | SUCCINATE-SEMIALDEHYDE-DEHYDROGENASE-RXN,GLYCOLALD-DEHYDROG-RXN,LACTALDDEHYDROG-RXN, | 61 |
| 49 | b1474 | fdnG | 4 | 2 | FORMATEDEHYDROG-RXN, | 100 |
| 50 | b1476 | fdnI | 4 | 2 | FORMATEDEHYDROG-RXN, | 100 |
| 51 | b1612 | fumA | 1 | 1 | FUMHYDR-RXN, | 159 |
| 52 | b1779 | gapA | 5 | 1 | GAPOXNPHOSPHN-RXN, | 823 |
| 53 | b2276 | nuoN | 4 | 2 | NADH-DEHYDROG-A-RXN, | 112 |
| 54 | b2277 | nuoM | 4 | 2 | NADH-DEHYDROG-A-RXN, | 112 |
| 55 | b2278 | nuoL | 4 | 2 | NADH-DEHYDROG-A-RXN, | 112 |
| 56 | b2279 | nuoK | 4 | 2 | NADH-DEHYDROG-A-RXN, | 112 |
| 57 | b2280 | nuoJ | 4 | 2 | NADH-DEHYDROG-A-RXN, | 112 |
| 58 | b2281 | nuoI | 4 | 2 | NADH-DEHYDROG-A-RXN, | 112 |
| 59 | b2282 | nuoH | 4 | 2 | NADH-DEHYDROG-A-RXN, | 112 |
| 60 | b2283 | nuoG | 4 | 2 | NADH-DEHYDROG-A-RXN, | 112 |
| 61 | b2284 | nuoF | 4 | 2 | NADH-DEHYDROG-A-RXN, | 112 |
| 62 | b2285 | nuoE | 1 | 5 | NADH-DEHYDROG-A-RXN, | 112 |
| 63 | b2287 | nuoB | 4 | 2 | NADH-DEHYDROG-A-RXN, | 112 |
| 64 | b2288 | nuoA | 4 | 2 | NADH-DEHYDROG-A-RXN, | 112 |
| 65 | b2296 | ackA | 5 | 1 | ACETATEKIN-RXN,PROPKIN-RXN, | 44 |
| 66 | b2779 | eno | 5 | 5 | 2PGADEHYDRAT-RXN, | 2865 |
| 67 | b2925 | fbaA | 5 | 1 | F16ALDOLASE-RXN, | 45 |
| 68 | b2926 | pgk | 5 | 5 | PHOSGLYPHOS-RXN, | 231 |
| 69 | b3236 | mdh | 1 | 1 | MALATE-DEH-RXN, | 187 |
| 70 | b3425 | glpE | 5 | 5 | THIOSULFATE-SULFURTRANSFERASE-RXN, | 782 |
| 71 | b3892 | fdoI | 4 | 2 | FORMATEDEHYDROG-RXN, | 100 |
| 72 | b3893 | fdoH | 1 | 1 | FORMATEDEHYDROG-RXN, | 100 |
| 73 | b3894 | fdoG | 1 | 1 | FORMATEDEHYDROG-RXN, | 100 |
| 74 | b3916 | pfkA | 5 | 1 | 6PFRUCTPHOS-RXN,NAD-KIN-RXN, | 182 |
| 75 | b3919 | tpiA | 5 | 5 | TRIOSEPISOMERIZATION-RXN, | 14 |
| 76 | b3956 | ppc | 5 | 5 | PEPCARBOX-RXN, | 187 |
| 77 | b4151 | frdD | 5 | 1 | R601-RXN,SUCC-FUM-OXRED-A-RXN, | 158 |
| 78 | b4152 | frdC | 5 | 1 | R601-RXN,SUCC-FUM-OXRED-A-RXN, | 158 |
| 79 | b4153 | frdB | 5 | 1 | R601-RXN,SUCC-FUM-OXRED-A-RXN, | 158 |
| 80 | b4154 | frdA | 5 | 1 | R601-RXN,SUCC-FUM-OXRED-A-RXN, | 158 |
| 81 | b1805 | fadD | 4 | 2 | ACYLCOASYN-RXN, | 112 |
| 82 | b2323 | fabB | 5 | 5 | 3-OXOACYL-ACP-SYNTH-BASE-RXN,3-OXOACYL-ACP-SYNTH-RXN,RXN0-2141,MALONYL-ACPDECARBOX-RXN, | 17 |
| 83 | b0207 | yafB | 5 | 5 | 25DKGR-RXN, | 3020 |
| 84 | b0221 | fadF | 1 | 1 | ACYLCOADEHYDROG-RXN, | 112 |
| 85 | b2308 | hisQ | 5 | 1 | ABC-14-RXN,ABC-3-RXN,ABC-37-RXN,ABC-4-RXN, | 123 |
| 86 | b2530 | iscS | 5 | 1 | RXN0-308, | 436 |
| 87 | b2676 | nrdF | 5 | 1 | RXN0-722,RXN0-747,RXN0-748,RXN0-1,RIBONUCLEOSIDE-DIP-REDUCTII-RXN, | 48 |
| 88 | b2904 | gcvH | 5 | 1 | GCVMULTI-RXN, | 67 |
| 89 | b2905 | gcvT | 5 | 1 | GCVMULTI-RXN,GCVT-RXN, | 67 |
| 90 | b2976 | glcB | 1 | 1 | MALSYN-RXN, | 1692 |
| 91 | b4014 | aceB | 5 | 1 | MALSYN-RXN, | 1692 |
| 92 | b4015 | aceA | 5 | 1 | ISOCIT-CLEAV-RXN, | 1689 |
| 93 | b4139 | aspA | 1 | 1 | ASPARTASE-RXN, | 6 |
| 94 | b4232 | fbp | 5 | 1 | F16BDEPHOS-RXN, | 235 |
| 95 | b0033 | carB | 5 | 1 | CARBPSYN-RXN, | 184 |
| 96 | b0888 | trxB | 5 | 5 | THIOREDOXIN-REDUCT-NADPH-RXN, | 520 |
| 97 | b0945 | pyrD | 5 | 5 | DIHYDROOROTOX-RXN, | 520 |
| 98 | b2234 | nrdA | 1 | 1 | ADPREDUCT-RXN,CDPREDUCT-RXN,GDPREDUCT-RXN,RXN0-1,RIBONUCLEOSIDE-DIP-REDUCTI-RXN,UDPREDUCT-RXN, | 151 |
| 99 | b2235 | nrdB | 1 | 1 | ADPREDUCT-RXN,CDPREDUCT-RXN,GDPREDUCT-RXN,RXN0-1,RIBONUCLEOSIDE-DIP-REDUCTI-RXN,UDPREDUCT-RXN, | 151 |
| 100 | b2476 | purC | 5 | 5 | SAICARSYN-RXN, | 6 |
| 101 | b2518 | ndk | 5 | 5 | CDPKIN-RXN,DADPKIN-RXN,DCDPKIN-RXN,DGDPKIN-RXN,DTDPKIN-RXN,DUDPKIN-RXN,GDPKIN-RXN,NUCLEOSIDE-DIP-KIN-RXN,UDPKIN-RXN, | 48 |
| 102 | b3831 | udp | 5 | 5 | URPHOS-RXN, | 793 |
| 103 | b4238 | nrdD | 1 | 1 | RXN0-723,RXN0-724,RXN0-746,RXN0-745,NRDACTMULTI-RXN,RIBONUCLEOSIDE-TRIP-REDUCT-RXN, | 48 |
| 104 | b1656 | sodB | 5 | 1 | SUPEROX-DISMUT-RXN, | 520 |
| 105 | b3908 | sodA | 1 | 1 | SUPEROX-DISMUT-RXN, | 520 |
| 106 | b3806 | cyaA | 5 | 1 | ADENYLATECYC-RXN, | 83 |
| 107 | b0314 | betT | 5 | 3 | TRANS-RXN-99, | 52 |
| 108 | b0336 | codB | 5 | 1 | TRANS-RXN-116, | 2707 |
| 109 | b0401 | brnQ | 5 | 1 | TRANS-RXN-126,TRANS-RXN-126A,TRANS-RXN-126B, | 4 |
| 110 | b0854 | potF | 5 | 1 | ABC-25-RXN, | 4477 |
| 111 | b0864 | artP | 5 | 1 | ABC-4-RXN, | 386 |
| 112 | b2309 | hisJ | 5 | 1 | ABC-14-RXN, | 231 |
| 113 | b2423 | cysW | 5 | 5 | ABC-70-RXN,ABC-7-RXN,ABC-7-RXN,ABC-70-RXN, | 7362 |
| 114 | b2425 | cysP | 5 | 5 | ABC-70-RXN,ABC-7-RXN, | 7362 |
| 115 | b2677 | proV | 5 | 1 | ABC-26-RXN, | 78 |
| 116 | b3453 | ugpB | 5 | 1 | ABC-34-RXN, | 902 |
| 117 | b3917 | sbp | 5 | 1 | ABC-7-RXN,ABC-70-RXN, | 7362 |
| 118 | b0621 | dcuC | 4 | 2 | TRANS-RXN-202,TRANS-RXN-202A, | 158 |
| 119 | b0963 | mgsA | 5 | 5 | METHGLYSYN-RXN, | 45 |
| 120 | b1033 | ycdW | 5 | 1 | |GLYOXYLATE-REDUCTASE-(NADP+)-RXN|,RXN0-300, | 52 |
| 121 | b2286 | nuoC | 4 | 2 | NADH-DEHYDROG-A-RXN, | 112 |
| 122 | b2747 | ispD | 5 | 1 | RXN0-277, | 184 |
| 123 | b3111 | tdcGa | 4 | 2 | LSERINEDEAM-RXN, | 100 |
| 124 | b3612 | yibO | 5 | 1 | 3PGAREARR-RXN, | 2865 |
| 125 | b3843 | yigC | 5 | 5 | 3-OCTAPRENYL-4-OHBENZOATE-DECARBOX-RXN, | 589 |
